# Advances in acupuncture modulation of signaling pathways for epilepsy treatment: A review

**DOI:** 10.1097/MD.0000000000046110

**Published:** 2025-11-28

**Authors:** Peiyao Meng, Xuan Wang, Wentao Yang, Yuxi Jiang, Weiping Cheng, Qi Zhang

**Affiliations:** a Graduate School, Heilongjiang University of Chinese Medicine, Harbin, Heilongjiang Province, China; b Department of Acupuncture and Moxibustion Department II, First Affiliated Hospital, Heilongjiang University of Chinese Medicine, Harbin, Heilongjiang Province, China; c Center for Preventive Treatment of Disease, First Affiliated Hospital, Heilongjiang University of Chinese Medicine, Harbin, Heilongjiang Province, China.

**Keywords:** acupoints, acupuncture, epilepsy, mechanism, signaling pathways

## Abstract

This study presents a comprehensive review and analysis of global research progress over the past 2 decades regarding the molecular mechanisms underlying acupuncture therapy for epilepsy. Our findings demonstrate that acupuncture, through single or combined acupoint stimulation (including but not limited to GV14, GV20, GV24, ST36, LI11, and ST37), exerts antiepileptic effects primarily by modulating key signaling pathways: PI3K/Akt, TLR4, NF-κB, mTOR, MAPK, BDNF/TrkB, and Nrf2/ARE/HO-1. The therapeutic mechanisms involve suppression of neuroinflammation, mitigation of oxidative stress, inhibition of neuronal apoptosis, regulation of neurotransmitter balance, and restoration of synaptic plasticity, collectively contributing to the reestablishment of excitation/inhibition homeostasis in the central nervous system. These findings establish an “acupoint-pathway-effect” regulatory network, highlighting acupuncture’s unique advantage as a multi-target, multi-level therapeutic approach for epilepsy management.

## 1. Introduction

Epilepsy is a chronic neurological disorder characterized by abnormal neuronal hyperexcitability and recurrent seizures, clinically manifesting as motor dysfunction, sensory disturbances, mood disorders, and autonomic dysregulation.^[1]^ With approximately 50 million affected individuals globally, epilepsy represents a significant public health burden, and its prevalence continues to rise.^[2]^ The pathogenesis of epilepsy involves a complex interplay of factors disrupting the excitatory/inhibitory balance in the CNS, including neuroinflammation, oxidative stress, immune dysregulation, neurotransmitter imbalances, ion channel dysfunction, and synaptic plasticity alterations.^[3]^ While the development of antiepileptic drugs has markedly improved seizure control for the majority of patients and substantially reduced the reliance on surgical interventions over recent decades, a significant unmet clinical need remains. Despite the availability of more than 15 anti-seizure drugs in clinical practice, approximately 30% of patients with epilepsy still respond inadequately to existing antiepileptic drugs, suggesting the presence of drug-resistant epilepsy.^[4]^ Moreover, long-term pharmacotherapy is often complicated by systemic side effects – such as cognitive impairment, drowsiness, and endocrine dysfunction – which considerably diminish quality of life and treatment adherence.^[5]^ These limitations highlight the need for alternative therapeutic strategies with improved safety profiles. Acupuncture, rooted in traditional Chinese meridian theory, has emerged as a promising complementary therapy for epilepsy. By modulating specific acupoints, acupuncture exerts multi-target regulatory effects with minimal side effects. Accumulating evidence demonstrates that acupuncture can restore neuronal homeostasis by simultaneously targeting multiple epilepsy-associated signaling pathways, offering a unique advantage over conventional single-target pharmacological approaches. This review systematically examines the pathological mechanisms underlying epilepsy-related signaling pathways and their modulation by acupuncture intervention.

## 2. Materials and methods

We conducted a literature search on RE using PubMed and China National Knowledge Infrastructure. The search terms included “epilepsy,” “acupuncture,” and “signaling pathways.” Relevant literature was screened through manual review. Studies published from 2005 to 2025 were included. Ultimately, 81 articles were included in the analysis.

Since this is a narrative review, ethical approval was not required.

## 3. The pathogenesis of epilepsy

### 3.1. Neuroinflammation and immune system activation in epileptogenesis

The development of epilepsy is closely associated with neuroinflammatory processes triggered by diverse CNS insults, including infections, traumatic brain injury, stroke, and neurodegenerative diseases. These pathological conditions activate microglia and astrocytes, leading to excessive production of pro-inflammatory cytokines such as interleukin-1β (IL-1β), interleukin-6 (IL-6), and tumor necrosis factor-α (TNF-α). These cytokines mediate epileptogenesis through dual mechanisms: by binding to neuronal and glial cell surface receptors, they acutely modulate ion channel function and synaptic transmission while simultaneously inducing long-term changes in synaptic plasticity through nuclear factor-κB (NF-κB)-mediated transcriptional regulation; and by promoting blood–brain barrier (BBB) dysfunction through matrix metalloproteinase activation, tight junction disruption, and leukocyte infiltration. Blood–brain barrier impairment results in albumin extravasation, which further activates astrocytes and establishes a self-perpetuating cycle of neuroinflammation and seizure activity. Importantly, this process is bidirectional – seizures exacerbate neuroinflammatory responses, while chronic inflammation lowers seizure thresholds, creating a vicious feedback loop.^[6,7]^

Acupuncture needle insertion induces a controlled, localized micro-mechanical injury, which elicits the release of damage-associated molecular patterns (DAMPs). These DAMPs are recognized by pattern recognition receptors expressed on resident immune cells, such as macrophages and mast cells, thereby initiating a mild, localized inflammatory response. Activated immune cells subsequently secrete cytokines and chemokines, which recruit additional immune cells to the acupuncture site. More importantly, this initial inflammatory cascade triggers 2 key downstream processes: first, it activates sensory nerve fibers, which modulate systemic immunity via neural reflex pathways and neurogenic inflammation; second, it drives the activation of endogenous inflammation resolution programs, thereby exerting efficient and ordered anti-inflammatory effects and promoting tissue repair.^[8]^

Key signaling pathways implicated in this neuroinflammatory cascade include phosphatidylinositol 3-kinase/protein kinase B (PI3K/Akt), toll-like receptor 4 (TLR4), NF-κB, c-Jun N-terminal kinase, p38 mitogen-activated protein kinase (p38 MAPK), brain-derived neurotrophic factor/tropomyosin receptor kinase B (BDNF/TrkB), and nuclear factor erythroid 2-related factor 2/antioxidant response element/heme oxygenase-1 (Nrf2/ARE/HO-1) pathways, all of which contribute significantly to epilepsy progression.

### 3.2. Neurotransmitter imbalances and ion channel dysfunction in epilepsy pathogenesis

The core pathophysiology of epilepsy stems from disrupted equilibrium between excitatory and inhibitory neurotransmission, primarily involving 2 key abnormalities: excessive glutamatergic excitation and deficient GABAergic inhibition. This dual dysregulation leads to pathological neuronal hypersynchronization and epileptiform activity.

Glutamate (Glu), the principal excitatory neurotransmitter, normally regulates neuronal excitability and cognitive function.^[9]^ However, pathological overactivation of glutamatergic signaling drives epileptogenesis through multiple mechanisms: sustained binding of excess synaptic glutamate to *N*-methyl-d-aspartate (NMDA) and α-amino-3-hydroxy-5-methyl-4-isoxazole propionic acid (AMPA) receptors causes abnormal calcium influx (not endocytosis), resulting in oxidative stress through free radical generation, mitochondrial dysfunction, and activation of caspase-dependent apoptotic pathways.^[10,11]^ These cascading events culminate in neuronal excitotoxicity and apoptosis, mediated through critical pathways including PI3K/Akt, TLR4, NF-κB, mTOR, MAPKs (JNK, p38 MAPK, ERK), and Nrf2/ARE/HO-1.

Conversely, γ-aminobutyric acid (GABA), the major inhibitory neurotransmitter, regulates neuronal activity through distinct receptor subtypes: GABA_A receptors mediate rapid inhibition via chloride influx, while GABA_B receptors induce slow inhibition through G-protein coupled modulation of potassium efflux and calcium influx.^[10]^ Dysfunction in either receptor system disrupts the delicate excitation/inhibition balance, lowering seizure thresholds and promoting epileptogenesis.

### 3.3. Synaptic plasticity and neural network remodeling in epileptogenesis

The epileptic brain exhibits profound alterations in synaptic connectivity and network organization, with hippocampal mossy fiber sprouting representing a hallmark pathological feature. This aberrant axonal reorganization establishes recurrent excitatory circuits within the dentate gyrus molecular layer, effectively lowering the seizure threshold of granule cells and creating a self-perpetuating cycle of hyperexcitability and spontaneous seizures.^[12]^ The formation of these abnormal synaptic connections through mossy fiber sprouting not only enhances neuronal synchronization but also reduces the capacity for adaptive network inhibition.

The interplay between epileptic activity and cognitive dysfunction involves complex modifications in synaptic plasticity mechanisms. As the neural substrate for learning and memory, synaptic plasticity normally maintains a delicate balance between long-term potentiation (LTP) and long-term depression (LTD).^[13]^ Physiological LTP, mediated by NMDA receptor activation and subsequent synaptic strengthening, supports memory formation, while LTD, dependent on α-amino-3-hydroxy-5-methyl-4-isoxazole propionic acid receptor internalization, enables adaptive network pruning. However, in the epileptic brain, this equilibrium shifts dramatically – excessive LTP combined with impaired LTD disrupts the excitation/inhibition ratio, lowers synchronization thresholds, and promotes maladaptive network reorganization.^[13,14]^

Acupuncture activates astrocytes and microglia, thereby promoting the release of BDNF and other neuromodulators. These factors subsequently modulate the function of NMDA receptors, a process that not only enhances synaptic efficacy but also helps maintain the dynamic balance between LTP and LTD. This cascade represents a key mechanism by which acupuncture regulates synaptic plasticity.^[15]^

Emerging evidence suggests that key signaling pathways including PI3K/Akt, NF-κB, mTOR, ERK, BDNF/TrkB, and Nrf2/ARE/HO-1 play pivotal roles in these pathological plasticity changes. Targeted modulation of these pathways may offer dual therapeutic benefits by simultaneously controlling seizure activity and mitigating associated cognitive deficits.^[16]^ The bidirectional relationship between epileptogenesis and synaptic remodeling underscores the importance of developing treatments that address both the seizure manifestations and their underlying network abnormalities.

### 3.4. Neural mechanisms of acupuncture: from peripheral stimulation to central modulation

The core mechanism underlying acupuncture’s therapeutic effects in human disease management is the regulation of local or distal physiological functions via the stimulation of specific acupoints on the body. This action is mediated by the “meridian” system, which is conceptualized as the pathway for the circulation of “Qi” – a term that can be interpreted as vital energy or an information-transmitting entity. During acupuncture, the induction of the “Deqi” sensation (typically described as soreness, numbness, distension, or heaviness) serves as a hallmark indicating that Qi has reached the pathological site and that the circulation of Qi and blood has been mobilized. Hui et al identified that the “deqi” sensation is a reflection of the activation of A-delta and C-fiber afferent neurons.^[17]^ By modulating the circulation of Qi and blood, acupuncture restores the “Yin-Yang” balance within the organism, thereby ultimately achieving the therapeutic objective.^[18]^

Although modern anatomy has not confirmed the physical existence of meridians, their long-distance regulatory effects may involve somatosensory autonomic reflex mechanisms. This reflex arc begins with the activation of peripheral sensory neurons in the dorsal root ganglia or trigeminal ganglia: sensory information is transmitted upward via the spinal cord to CNS for integration, which then triggers the autonomic nervous system to regulate peripheral organs, ultimately achieving extensive modulation of various physiological functions.^[19]^ Specifically, signals generated by the activation of skin receptors at acupoints and surrounding tissues (including neural impulses induced by acupuncture, tactile stimulation, and pain) are first transmitted to the dorsal horn of the spinal cord or the spinal trigeminal nucleus of the medulla oblongata via Aβ, Aδ, and C fibers. Among these signals, those originating from the trunk and extremities can ascend to the thalamus either directly via the spinothalamic tract or indirectly via the spinoreticulothalamic tract; in contrast, signals from the head and face (which are mostly innervated by the trigeminal nerve) are first conveyed to the trigeminal nucleus and then reach the thalamus through the trigeminothalamic tract. Additionally, a considerable portion of acupuncture-induced impulses can ascend to the limbic cortex via the spinoneural circuit, spinoreticular tract, and spinocerebellar tract, while a higher proportion of tactile and pain-induced impulses tend to be transmitted to the sensorimotor cortex through the spinothalamic tract. Eventually, the thalamus further relays the relevant information to the cerebral cortex.^[20,21]^

For instance, when electroacupuncture with low-current and low-frequency parameters is applied to stimulate acupoints Jianshi (PC5) – Neiguan (PC6), Zusanli (ST36) – Shangjuxu (ST37), it acts directly on the median nerve beneath acupoint PC5-6 and the lateral sural cutaneous nerve adjacent to acupoint ST36-37. This stimulation is followed by the activation of cells in the arcuate nucleus of the hypothalamus, the ventrolateral periaqueductal gray of the midbrain, and the rostral reticular formation of the medulla oblongata. This activation exerts 2 key effects: on 2 hand, it inhibits cardiovascular sympathetic neurons, and on the other hand, it further reduces the activity of premotor sympathetic neurons in the rostral ventrolateral medulla.^[22]^ Another study demonstrated that electroacupuncture stimulation at the “Dazhui” acupoint (GV14) markedly suppressed pentylenetetrazol (PTZ)-induced epileptiform activity in neurons of the ventrobasal thalamus, and it was able to nearly abolish PTZ-induced epileptiform discharges.^[23]^

**Figure 1.**
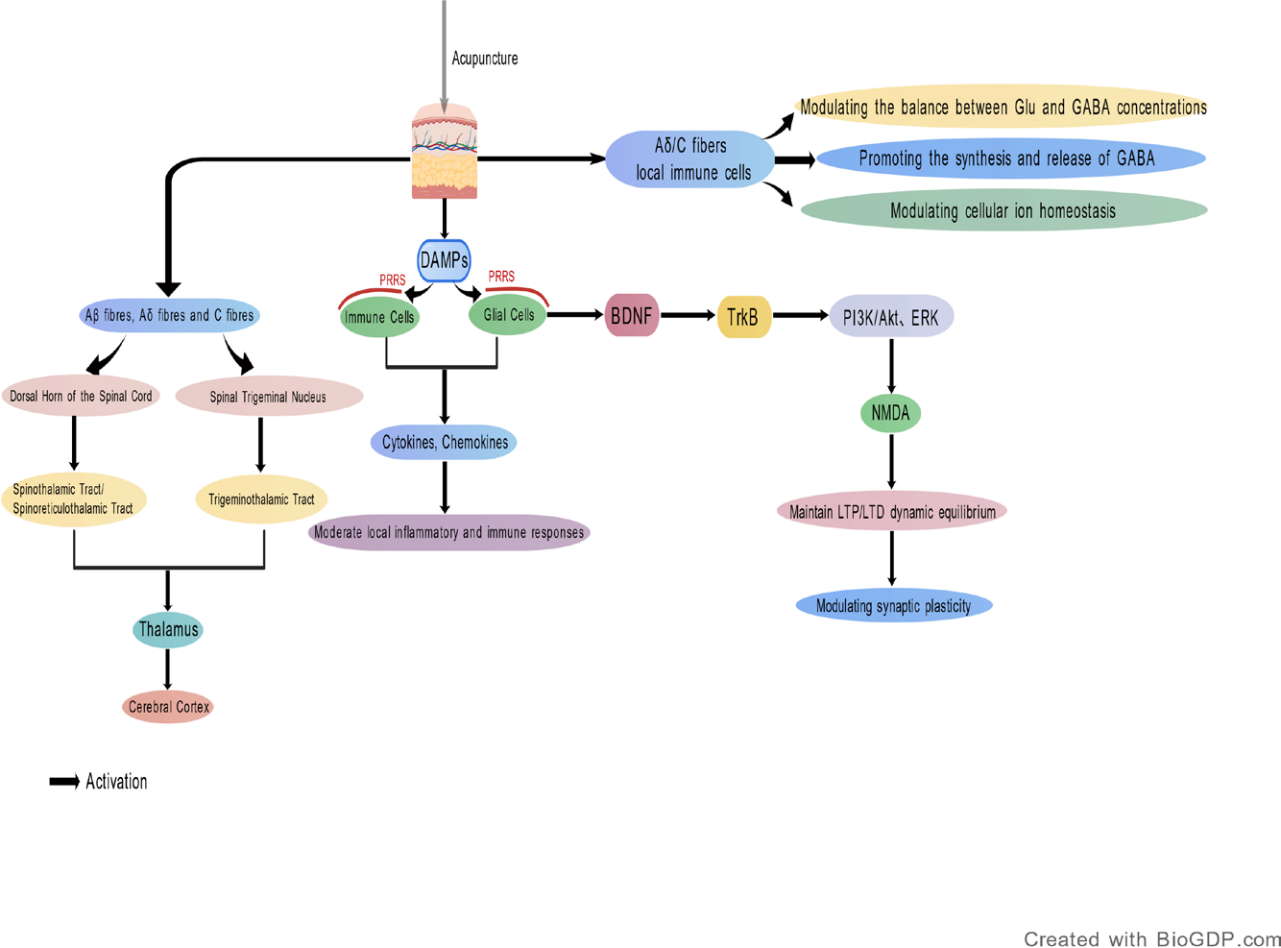
The pathogenesis of epilepsy.

In summary, the mechanism underlying the therapeutic effect of acupuncture on epilepsy may involve multi-level regulatory processes. On one hand, acupuncture modulates neural conduction in the thalamus and cortical regions through peripheral nerve stimulation, thereby regulating epileptiform discharges. On the other hand, its effects also involve alterations in inflammatory responses, neurotransmitter metabolism, and synaptic plasticity, as well as the involvement of complex signaling pathways at the transcriptional level, ultimately promoting neural remodeling (Fig. 1). Given its regulatory potential across multiple targets and pathways, acupuncture is expected to become a promising therapeutic strategy for epilepsy.

## 4. Epilepsy-related signaling pathways

### 4.1. The PI3K/Akt signaling pathway

#### 4.1.1. *The PI3K/Akt signaling pathway in epilepsy pathogenesis and treatmen*t

The phosphatidylinositol 3-kinase/protein kinase B (PI3K/Akt) pathway serves as a crucial regulator of cell survival, metabolism, and growth, playing a multifaceted role in epilepsy development and progression. Structurally composed of p110 catalytic and p85 regulatory subunits, PI3K becomes activated through various membrane receptors including GPCRs, RTKs, and IGF-1Rs. This activation triggers the conversion of PIP2 to PIP3, which subsequently recruits Akt to the plasma membrane via its PH domain, leading to Akt phosphorylation at Thr308 and Ser473. Once activated, phospho-Akt orchestrates diverse cellular responses primarily through mTOR and GSK-3β regulation.

Within epileptic hippocampi, PI3K/Akt activation exerts neuroprotective effects by: modulating apoptotic transcription factors, upregulating antiapoptotic Bcl-2 family proteins, and suppressing caspase-3/9 activation.^[24]^ These mechanisms are particularly relevant for vulnerable CA1 and CA3 pyramidal neurons, with experimental evidence demonstrating that PI3K/Akt signaling enhancement reduces both neuronal apoptosis and oxidative stress while suppressing seizure activity.^[25]^

Beyond its antiapoptotic role, the PI3K/Akt pathway significantly influences epilepsy-associated neuroinflammation and cognitive dysfunction. PTZ-induced epileptic models show concurrent PI3K/Akt activation and neuroinflammation, characterized by elevated hippocampal IL-1β expression and impaired synaptic plasticity. Notably, IL-1β receptor blockade reduces the p-Akt/Akt ratio while restoring cognitive function, suggesting inflammatory mediators disrupt cognition through this pathway.^[26]^ Emerging therapeutic approaches like electroacupuncture demonstrate promising results by activating microglial PI3K/Akt signaling, thereby enhancing BDNF expression and promoting synaptic plasticity.^[27]^ These findings highlight the PI3K/Akt pathway as a pivotal intersection point for multiple epileptogenic processes, warranting further investigation into its therapeutic potential.

#### 4.1.2. Mechanisms of acupuncture in modulating the PI3K/Akt pathway for epilepsy treatment

Growing evidence demonstrates that acupuncture exerts neuroprotective effects against epilepsy through targeted activation of the PI3K/Akt signaling pathway. Yang et al systematically investigated this mechanism using both pharmacological and acupuncture interventions.^[28]^ Their key findings revealed that pretreatment with specific PI3K/Akt inhibitors substantially diminished the protective effects of Baihui (GV20) and GV14 acupuncture, as evidenced by a reduction in CA3 pyramidal cell survival and an increase in neuronal damage markers compared to acupuncture-only treatment. Importantly, acupuncture monotherapy produced significant neuroprotection, increasing PI3K expression and enhancing neuronal viability in hippocampal CA1/CA3 regions relative to untreated epileptic models. These effects are mediated through PI3K/Akt-dependent inhibition of apoptosis cascades, reducing nerve damage secondary to epilepsy.

Further studies have expanded these findings to different acupuncture modalities and acupoint combinations. Electroacupuncture stimulation at bilateral Hegu (LI4) and Taichong (LR3) acupoints has been shown to effectively inhibit hippocampal neuronal apoptosis through activation of the PI3K/Akt signaling pathway.^[29]^ This therapeutic intervention significantly upregulates the expression of antiapoptotic protein Bcl-2 while concurrently downregulating pro-apoptotic Bax in hippocampal tissues. The coordinated modulation of these apoptosis-related proteins suggests that electroacupuncture exerts its neuroprotective effects by rebalancing the hippocampal apoptotic cascade through PI3K/Akt pathway activation.

Similarly, another experiment shows that electroacupuncture stimulation at unilateral ST36 and Quchi (LI11) acupoints was found to significantly enhance PI3K/Akt signaling pathway activity in the rat cerebral cortex.^[30]^ This intervention demonstrated marked antiapoptotic effects, characterized by an elevated Bcl-2/Bax ratio and reduced caspase-3 expression compared to control animals.

These molecular changes collectively suggest that acupuncture functions as a multi-target therapy that directly activates the PI3K/Akt signaling pathway, rebalances apoptotic regulators, and mitigates secondary neuronal injury.

### 4.2. The TLR4 signaling pathway

#### 4.2.1. The TLR4 signaling pathway in epileptogenesis and neuroinflammation

The toll-like receptor 4 (TLR4) pathway serves as a critical mediator between neuroinflammatory processes and epilepsy development. As a key pattern recognition receptor, TLR4 responds to both pathogen-associated and DAMPs through myeloid differentiation factor 88-dependent and independent signaling cascades. In the CNS, TLR4 activation in microglia, astrocytes, and neurons triggers downstream NF-κB and MAPK pathways, leading to increased expression of pro-inflammatory cytokines including IL-1β, IL-6, and TNF-α.^[31]^

Experimental evidence demonstrates the pathway’s direct involvement in epileptogenesis. Chronic administration of sodium valproate and carbamazepine in lithium-pilocarpine rat models significantly elevated hippocampal and amygdalar expression of TLR4, NF-κB, IL-1β, and TNF-α at both mRNA and protein levels compared to untreated epileptic controls. Conversely, TLR4-deficient models showed markedly reduced expression of these inflammatory markers in the CA3 hippocampal subregion and amygdala, confirming TLR4’s regulatory role in epilepsy-associated neuroinflammation.^[32]^ Maroso et al concluded that TLR4 receptor antagonists not only control the development of epilepsy but also reduce recurrent acute and chronic seizures.^[33]^

Mechanistic studies further reveal TLR4’s involvement in apoptotic regulation during epileptic progression. In kainic acid (KA)-induced models, TLR4 pathway inhibition significantly reduced hippocampal apoptosis, evidenced by decreased Bax and caspase-3 expression alongside increased Bcl-2 levels. These molecular changes suggest that TLR4 pathway inhibition may exert neuroprotective effects by modulating apoptotic factor levels.^[34]^

#### 4.2.2. Acupuncture modulation of TLR4 signaling in epilepsy management

Experimental studies showed that TLR4 expression was significantly elevated in the prefrontal cortex, hippocampus and cerebral cortex in a rat model of the KA-induced epilepsy, and the overexpressed TLR4 exacerbated the neuroinflammation through the following pathways: on the one hand, it directly activated the NF-κB signaling pathway to promote the transcriptional expression of pro-inflammatory factors; on the other hand, it mediated the activation of the MAPK signaling pathway through the Ca^2+^ influx. However, electrical stimulation by auricular acupuncture (using clip electrodes, with the cathode at the ear apex and anode at the ear lobe) could effectively reduce the TLR4 expression level while inhibiting the transduction of 2 key inflammatory signaling pathways, NF-κB and MAPK, and attenuating neuronal inflammatory injury. The results of this experiment suggest that auricular acupuncture electrical stimulation may play an anti-inflammatory role by regulating the TLR4 signaling pathway, effectively controlling epileptic seizures.^[35]^

### 4.3. The NF-κB signaling pathway

#### 4.3.1. The dual role of NF-κB signaling in epilepsy pathogenesis and treatment

The nuclear factor-kappa B (NF-κB) pathway serves as a critical regulator in epilepsy, exhibiting complex dual roles in both neuroprotection and neuroinflammation. As a ubiquitous eukaryotic transcription factor, NF-κB mediates diverse cellular processes including inflammatory responses, neuronal survival, and apoptotic regulation through 2 distinct activation pathways.^[36]^ The canonical pathway involves IKKβ-mediated IkBα phosphorylation and subsequent RelA/p50 nuclear translocation, while the noncanonical pathway relies on IKKα-dependent processing of p100 to p52, forming RelB/p52 complexes that enter the nucleus.^[37]^

Comprehensive studies have shown that the NF-κB signaling pathway exhibits complex bidirectional regulation in epilepsy. Singh et al demonstrated transient NF-κB activation post-seizure upregulates antiapoptotic Bcl-2, suggesting potential neuroprotective effects.^[38]^ Conversely, Liu et al observed that sustained NF-κB activation promotes hippocampal neuron apoptosis, exacerbates neuroinflammation, and increases seizure frequency and duration.^[39]^ This functional dichotomy appears mediated by differential subunit phosphorylation – p52 modification enhances Bcl-2 expression, p65-Ser529 phosphorylation supports cell growth, while other p65 phosphorylation sites may drive pro-apoptotic and inflammatory responses.^[40,41]^

The pathway’s inflammatory role is further evidenced in PTZ-induced models, where interleukin-1 receptor-associated kinase 4 activates NF-κB to promote NLRP3 inflammasome transcription, triggering pyroptosis and seizure recurrence.^[42]^ The M1 microglia mainly secretes pro-inflammatory cytokines and exacerbates oxidative stress, whereas the M2 microglia mainly secretes anti-inflammatory cytokines and promotes tissue repair.^[43]^ Thus, facilitating the shift in microglia phenotype from M2 to M1 is critical in the treatment of neurological disorders. Studies showed that inhibition of the NF-κB signaling pathway may trigger microglia M2 phenotype polarization and promote tissue repair and regeneration. It may also inhibit hippocampal mossy fiber germination to control seizures and exert neuroprotective effects.^[44,45]^

#### 4.3.2. Acupuncture modulation of NF-κB signaling in epilepsy treatment

**Figure 2.**
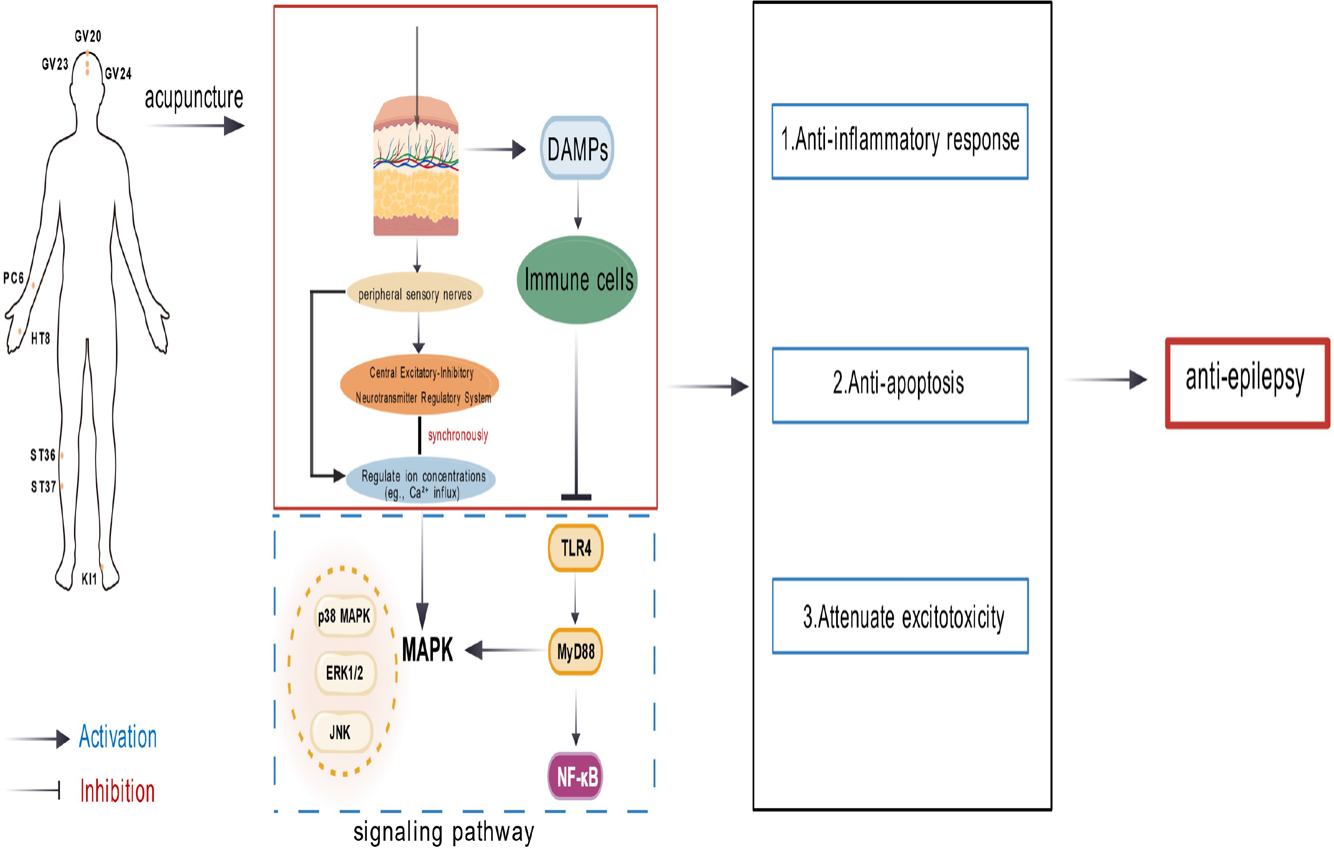
A mechanistic diagram depicting the regulation of TLR4, NF-κB, and MAPK signaling pathways by acupuncture. MAPK = mitogen-activated protein kinase, NF-κB = nuclear factor-κB, TLR4 = toll-like receptor 4.

Acupuncture induces controlled micro-mechanical injury at the local tissue site, activating peripheral afferent nerve fibers that convey injury-associated signals to the CNS. In response, the CNS modulates TLR4-mediated pattern recognition signaling – TLR4 being a member of the PRR family.^[46]^ This modulation leads to suppression of the downstream myeloid differentiation primary response 88 (MyD88)-dependent signaling cascade and impedes nuclear translocation of NF-κB, ultimately resulting in the downregulation of pro-inflammatory effector molecules (Fig. 2). Experimental studies demonstrate that acupuncture exerts significant anti-inflammatory and neuroprotective effects in epilepsy through targeted regulation of NF-κB signaling. Xie et al revealed that electroacupuncture at GV20 effectively downregulates NF-κB pathway activity, resulting in a marked increase in anti-inflammatory cytokines alongside decreased pro-inflammatory mediators in rat models.^[47]^ This cytokine rebalancing correlates with suppressed glial cell activation and reduced neuronal damage, suggesting NF-κB inhibition as a key mechanism underlying acupuncture’s neuroprotective properties.

The study showed that electroacupuncture stimulation of GV20 and Shenting (GV24) acupoints may exert anti-neuronal apoptosis effects by inhibiting the NF-κB signaling pathway and significantly reducing the expression of pro-apoptotic factors Bax and Fas. Meanwhile, electroacupuncture intervention effectively improved cognitive dysfunction in rats, and its neuroprotective effect may be related to the reduction of neuronal apoptosis.^[48]^ These findings suggest a dual therapeutic mechanism where acupuncture simultaneously: attenuates inflammatory damage through NF-κB suppression and preserves neuronal viability by rebalancing apoptotic regulators. The observed cognitive improvements further support acupuncture’s potential for addressing both seizure control and epilepsy-associated neurological deficits.

### 4.4. The mTOR signaling pathway

#### 4.4.1. The mTOR signaling pathway in epilepsy: mechanisms and therapeutic implications

The mammalian target of rapamycin (mTOR) pathway represents a crucial regulator of neuronal excitability and synaptic plasticity, playing dual roles in both normal brain function and epileptogenesis. As an atypical serine/threonine kinase, mTOR forms 2 functionally distinct complexes: mTORC1, which primarily regulates protein synthesis and autophagy inhibition, and mTORC2, involved in cytoskeletal organization and synaptic maintenance.^[49]^ These complexes integrate upstream signals, particularly from the PI3K/Akt signaling pathway induced by growth factors and their superfamily, to coordinately modulate essential neuronal processes including excitation/inhibition balance, synaptic plasticity, cognitive function and cellular autophagy.^[13,47,50]^ While physiological mTOR activity supports cortical development and memory formation, pathological hyperactivation disrupts neuronal homeostasis. Excessive mTOR signaling promotes aberrant synaptic proliferation and neuronal hyperexcitability, increasing seizure susceptibility.^[51]^

It has been found that mTOR is a negative regulator of autophagy, and inhibition of the mTOR signaling pathway activates autophagy, regulates the balance between GABA and Glu to control epilepsy development, and serves to reduce the severity of seizures.^[52]^ Another experiment investigated the dynamic changes of autophagy-related factor expression in the hippocampus of status epilepticus (SE) rats. The high expression of p-PRAS40 was found to activate the mTOR pathway and decrease the level of cellular autophagy.^[53]^ Similarly, the Rac1 protein, which is negatively regulated by the mTOR pathway, has been implicated in epileptogenesis.^[54]^ Activated Rac1 protein can achieve antiepileptic effects by inhibiting neurons and upregulating autophagic links. The tuberous sclerosis complex (TSC) is an important protein complex in the mTOR signaling pathway that negatively regulates mTORC1 activity. It was found that deletion of Tsc1 in a rat model leads to upregulation of the mTOR pathway in hippocampal neurons and increases the number of synapses on postsynaptic neurons as well as enhances Glu function.^[55]^ Rapamycin is an inhibitor of the mTOR pathway. The results of experimental studies showed that the application of rapamycin in a rat model of KA-induced epilepsy could block the abnormal activation of the mTOR signaling pathway. This reduces mossy fiber outgrowth, neuronal apoptosis, and the incidence of spontaneous epilepsy.^[56]^ These findings highlight mTOR’s central position in epileptogenesis and its potential as a target for novel treatments addressing both seizure control and associated cognitive comorbidities.

#### 4.4.2. Acupuncture modulation of mTOR signaling in epilepsy treatment

Experimental studies demonstrate that acupuncture exerts antiepileptic effects through bidirectional regulation of the mTOR signaling pathway. Temporal lobe epilepsy rats receiving 2-week electroacupuncture treatment at GV14 and GV20 acupoints showed significantly decreased phosphorylated mTOR levels in hippocampal CA3 and dentate gyrus regions, accompanied by increased expression of autophagy markers.^[57]^ These findings suggest acupuncture may suppress seizure activity by inhibiting mTOR overactivation and promoting neuronal autophagy to maintain cellular homeostasis. Further evidence reveals acupuncture’s capacity for context-dependent mTOR modulation. Stimulation of GV20, Shuigou (GV26), Fengfu (GV16) through Yamen (GV15), and bilateral LI4 acupoints bidirectionally regulated cortical autophagy markers in epileptic models.^[58]^

Interestingly, acupuncture at Fengchi (GB20), GV16 and GV14 produced opposite effects, activating mTOR signaling while simultaneously downregulating NLRP3 inflammasome components and pro-inflammatory cytokines.^[59]^ This dual regulatory capability highlights acupuncture’s unique advantage in selectively modulating the mTOR pathway based on specific pathological conditions.

### 4.5. The MAPK signaling pathway

#### 4.5.1. The MAPK signaling pathway in neurological disorders and epilepsy

The MAPK family, comprising 3 major subfamilies – p38 MAPK, extracellular signal-regulated kinase (ERK), and c-Jun N-terminal kinase – represents a crucial group of serine/threonine kinases that regulate fundamental cellular processes including proliferation, differentiation, and programmed cell death. These evolutionarily conserved signaling cascades have been increasingly recognized for their pivotal roles in various neurological disorders, particularly in epilepsy pathogenesis. Through their ability to integrate diverse extracellular stimuli into coordinated intracellular responses, MAPKs modulate neuronal excitability, synaptic plasticity, and inflammatory responses, all of which contribute significantly to epileptogenesis and seizure propagation.

##### 4.5.1.1. The p38 MAPK and JNK signaling pathways in epilepsy pathogenesis

The p38 MAPK and JNK pathways play critical roles in regulating apoptosis, neuroinflammation, and hippocampal neuronal damage in epilepsy. Experimental evidence demonstrates that pharmacological modulation of these pathways produces significant neuroprotective effects. Ganoderic acid A treatment in epileptic rat models effectively downregulated phosphorylated p38 and JNK protein levels, concomitant with reduced expression of apoptotic mediators caspase-3 and Bax, ultimately inhibiting neuronal apoptosis.^[60]^ Similarly, the synthetic triterpenoid CDDO-ME exhibited potent anti-inflammatory effects in SE models by suppressing p38 MAPK phosphorylation, which subsequently inhibited microglial activation and reduced TNF-α production.^[61]^ Furthermore, p38 MAPK inhibition upregulated excitatory amino acid transporter 2 expression, enhancing glutamate clearance and preventing seizure generation caused by excitatory amino acid accumulation.^[62]^

The JNK pathway, comprising 3 isoforms (JNK1-3), shows distinct regional distribution within the hippocampus, with JNK3 present throughout hippocampal regions while JNK1 localized specifically to CA3, CA4, and dentate gyrus.^[63]^ This anatomical specificity correlates with functional importance in epileptic brain injury, as demonstrated by JNK1 or JNK3 knockout mice exhibiting remarkable neuroprotection following KA exposure. Specifically, this was manifested as reduced neuronal degeneration, downregulation of apoptosis-related gene expression, decreased glial reactivity, and attenuated excitatory neurotoxicity.^[64]^ These findings collectively establish both p38 MAPK and JNK pathways as promising therapeutic targets for addressing multiple pathological components of epilepsy.

##### 4.5.1.2. The neuroprotective role of ERK signaling in epilepsy pathogenesis

Within the MAPK family, the ERK pathway demonstrates functionally distinct characteristics compared to its JNK and p38 MAPK counterparts. While JNK and p38 activation typically promote apoptotic processes and exacerbate neuronal injury, ERK signaling exhibits neuroprotective properties that facilitate neural repair mechanisms. The ERK pathway contributes to epilepsy modulation through multiple mechanisms, including regulation of apoptotic pathways, ferroptosis, LTP, and cognitive function.^[65,66]^

Experimental evidence highlights ERK’s therapeutic potential in epilepsy management. Zhang et al demonstrated that ginsenoside Rg1 administration in lithium chloride-induced epileptic rats modulated ERK signaling, resulting in significant antiapoptotic effects accompanied by reduced seizure duration and frequency, along with improved learning and memory performance.^[67]^ Another study found that ERK pathway inhibition in epileptic mouse models attenuated seizure activity by modulating excitatory neuronal ferroptosis and oxidative stress.^[68]^ These findings collectively suggest that targeted ERK pathway modulation may offer dual benefits for both seizure control and cognitive preservation in epilepsy.

#### 4.5.2. Acupuncture modulation of MAPK signaling pathways in epilepsy treatment

Accumulating evidence demonstrates that acupuncture exerts antiepileptic effects through targeted regulation of MAPK signaling pathways. Stimulation of GV20, PC6, and Yongquan (KI1) acupoints significantly suppresses p38 MAPK activation, resulting in a reduction in hippocampal microglial activation and decrease in pro-inflammatory cytokine levels (IL-1β, TNF-α), which correlates with improved neurological function and restored cognitive performance in epileptic rats.^[69]^ Experimental studies showed that 2 Hz electroacupuncture stimulation of the periauricular area and the ST36 and Shangjuxu (ST37) points in epileptic rats effectively inhibited the abnormal discharges in the CA1 area of the hippocampus and significantly reduced the level of ERK1/2 phosphorylation. This result suggests that electroacupuncture may exert antiepileptic effects by inhibiting the activity of ERK1/2 signaling pathway.^[70]^ Experimental studies confirmed that acupuncture at GV16 and Shangxing (GV23) points significantly improved hippocampal synaptic plasticity and attenuated hippocampal tissue inflammatory injury. Meanwhile, acupuncture inhibited the hippocampal apoptosis pathway by activating the ERK signaling pathway, thereby restoring cognitive function and protecting hippocampal neural survival in rats.^[71]^

The JNK/c-Jun axis represents another key target, with bilateral Shaofu (HT8) acupuncture demonstrating increased GABAergic neurotransmission, downregulation of c-Jun expression, and reduction in JNK phosphorylation. These changes collectively attenuate KA-induced excitotoxicity and provide neuroprotection.^[72]^ The ability to differentially modulate distinct MAPK pathways underscores acupuncture’s unique potential as a multi-target therapy for epilepsy management.

### 4.6. The BDNF/TrkB signaling pathway

#### 4.6.1. The dual role of BDNF/TrkB signaling in epilepsy pathogenesis

The brain-derived neurotrophic factor (BDNF) and its high-affinity receptor tropomyosin receptor kinase B (TrkB) constitute a critical signaling system abundantly expressed in hippocampal and cortical regions, primarily secreted by neurons and astrocytes. This pathway regulates fundamental neurological processes including synaptic plasticity, excitation/inhibition balance, and cognitive function through specific ligand–receptor interactions.^[73]^ Beyond its neurotrophic functions, BDNF/TrkB signaling significantly modulates neuroinflammatory responses, playing a complex role in epilepsy pathophysiology.^[74]^

Current research reveals a paradoxical dual role for BDNF in epileptogenesis. While some studies report anticonvulsant effects through neuroprotective mechanisms, substantial evidence implicates BDNF as a pro-epileptic factor.^[75,76]^ The initial post-seizure upregulation of BDNF/TrkB in the CNS likely represents an endogenous protective response.

In the early stage of epileptic seizures, the widespread increased expression of BDNF and its receptors in the CNS is an endogenous protective response. However, an acute and sustained increase in BDNF levels may lead to epilepsy by neuronal overexcitation through mossy fiber budding, and chronic treatment with BDNF can induce synaptic changes that promote neuronal survival and serve to protect the nervous system. It was found that BDNF/TrkB mRNA expression was significantly reduced after injection of antiepileptic drugs into the epileptic mouse model. The results of this experiment suggest that by downregulating the BDNF/TrkB signaling pathway may alleviate the inflammatory response in epileptic mice, reduce the degree of hippocampal cell damage, and improve cognitive ability.^[77]^ It was found that the BDNF/TrkB axis is highly upregulated in the hippocampus in animal models of epilepsy, and this upregulation induces downregulation of K⁺-Cl⁻ cotransporter KCC2 in GABAergic neurons, leading to neuronal hyperexcitability and spontaneous recurrent seizures in mice.^[73,78]^ Andreska et al demonstrated through experimental studies that BDNF is abundantly expressed presynaptically at hippocampal glutamatergic synapses, suggesting that the elevated neural excitability during seizures may be related to the release of BDNF.^[79,80]^ These findings suggest that precise modulation of BDNF/TrkB signaling – rather than complete inhibition may represent an optimal strategy for epilepsy treatment, balancing neuroprotection against hyperexcitability risks.

#### 4.6.2. Acupuncture modulation of BDNF/TrkB signaling in epilepsy treatment

**Figure 3.**
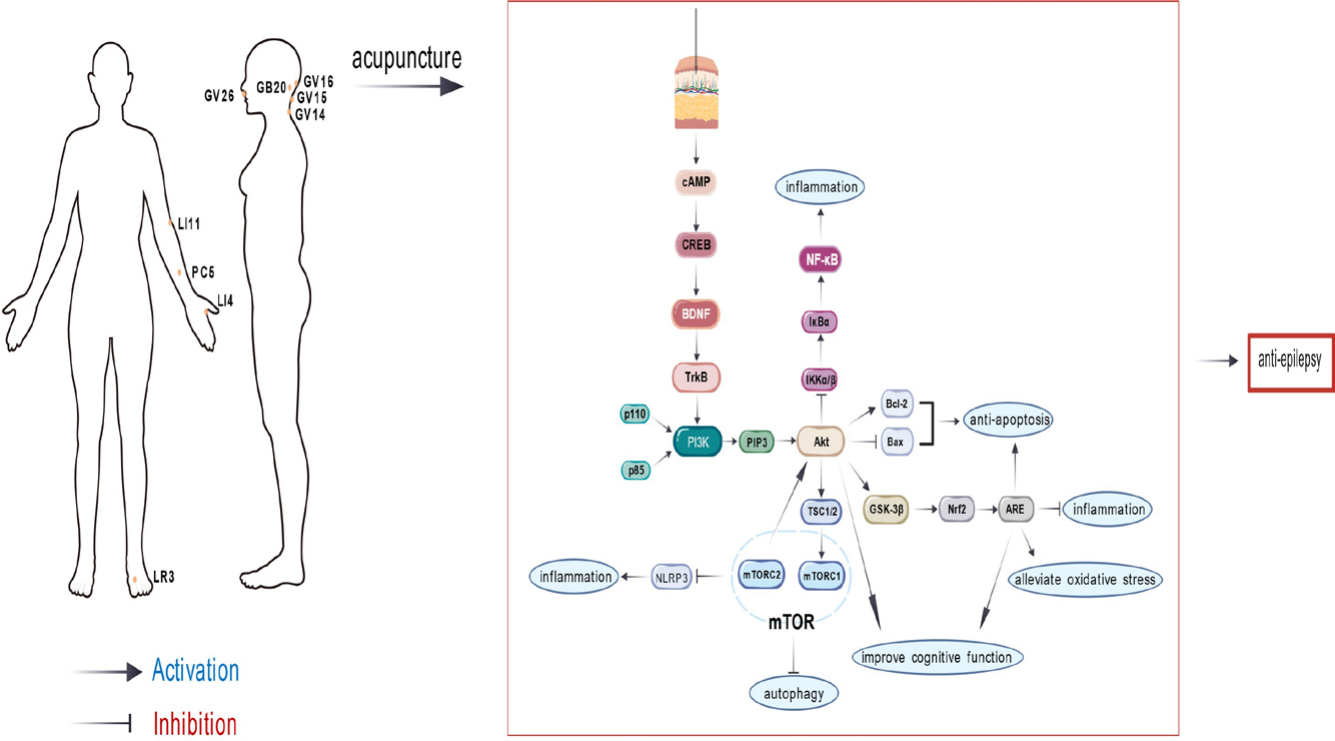
A mechanistic diagram depicting the regulation of PI3K/Akt, mTOR, BDNF/TrkB, and Nrf2/ARE/HO-1 signaling pathways by acupuncture. ARE = antioxidant response element, BDNF = brain-derived neurotrophic factor, HO-1= heme oxygenase-1, mTOR = mammalian target of rapamycin, Nrf2 = nuclear factor erythroid 2-related factor 2, TrkB = tropomyosin receptor kinase B.

Acupuncture stimulation activates the cAMP signaling pathway, leading to the phosphorylation of the CREB protein. The phosphorylated CREB then binds to the cAMP response element on target genes, initiating transcription and thereby promoting the synthesis of downstream BDNF.^[81]^ It was found that acupuncture at GV20 points may significantly reduce the level of apoptosis in the hippocampal CA1 region by up-regulating BDNF, and protect hippocampal neural survival while improving the learning and memory abilities of rats.^[82]^ Furthermore, electroacupuncture at ST36 and LI11 produces multi-modal therapeutic benefits through BDNF/TrkB activation: up-regulating the expression of related synaptic proteins, restoring the synaptic structural plasticity of hippocampal neurons, and improving the learning and memory deficits of the rats. At the same time, it reduced brain damage and neuronal apoptosis and had a protective effect on hippocampal neurons.^[83]^ The ability to simultaneously enhance neurotrophic support while reducing apoptotic damage highlights acupuncture’s unique potential as a dual-target therapy for epilepsy-related neurological deficits (Fig. 3).

### 4.7. The Nrf2/ARE/HO-1 signaling pathway

#### 4.7.1. The Nrf2/ARE/HO-1 signaling pathway in epilepsy: a key antioxidant defense mechanism

The nuclear factor erythroid 2-related factor 2/antioxidant response element/heme oxygenase-1 (Nrf2/ARE/HO-1) pathway serves as a crucial cellular defense system against oxidative stress, inflammation, and apoptosis, playing a pivotal role in maintaining neuronal homeostasis.^[84]^ In the physiological state, the transcription factor Nrf2 is inactivated by binding to the repressor protein Keap1 in the cytoplasm.^[85]^ When oxidative stress occurs, excess reactive oxygen species disrupt the Keap1–Nrf2 interaction, prompting Nrf2 to translocate to the nucleus and bind to the ARE in the promoter region of target genes, which in turn activates the transcription of several cytoprotective genes, including HO-1.^[86]^ HO-1 collectively exerts antioxidant and anti-inflammatory effects by catalyzing the degradation of heme to generate biliverdin, carbon monoxide, and abile iron, where biliverdin is further converted to the potent antioxidant bilirubin.^[87]^ Furthermore, this pathway demonstrates cross-talk with NF-κB signaling, enabling broader modulation of neuroinflammatory processes relevant to epilepsy and neurodegeneration.^[88]^

The study revealed KA-induced specific activation of the Nrf2 signaling pathway in SE rats.^[85]^ Nrf2 mRNA levels were significantly elevated in the hippocampus of rats 24 hours after SE and persisted for 2 weeks, whereas only a transient slight increase was seen in the cerebral cortex. Second, at the protein level, the hippocampus of rats in the chronic epileptic phase showed upregulated protein expression of Nrf2 and its target genes. In addition, immunohistochemical analysis showed that Nrf2 expression was mainly enriched in neurons and astrocytes in the CA1 region of the hippocampus, while no significant changes were observed in the CA3 region and cortex. These results confirmed that the Nrf2 pathway was activated by region-specific (hippocampal CA1 region predominantly), time-dependent (mRNA elevation in the acute phase and protein expression in the chronic phase) and cell-selective (neurons and astrocytes) activation after SE, suggesting that it is involved in the spatio-temporal specific regulation of epileptic pathology and exerts antioxidant effects.

Other studies have confirmed that the Nrf2/ARE signaling pathway can be regulated through multiple mechanisms to exert neuroprotective effects.^[88]^ Stimulation of the Nrf2/ARE signaling pathway upregulated the expression of the antiapoptotic factor Bcl-2 and downregulated the expression of the pro-apoptotic factors Bax and caspase-3, which significantly inhibited neuronal apoptosis and alleviated the neuronal damage-associated with seizures. At the same time, stimulation of this signaling pathway can inhibit the expression of NF-κB protein and pro-inflammatory cytokines, reducing the inflammatory response, thereby reducing the severity of seizures.

#### 4.7.2. Acupuncture modulation of Nrf2/ARE/HO-1 signaling in epilepsy treatment

Emerging evidence demonstrates that acupuncture exerts neuroprotective effects in epilepsy through bidirectional regulation of the Nrf2/ARE/HO-1 pathway. Experimental studies reveal that electroacupuncture at ST36 produces context-dependent modulation, with some protocols showing significant suppression of HO-1 and Nrf2 expression, effectively controlling oxidative stress in epileptic models.^[89]^ Conversely, alternative approaches combining GV20 and ST36 stimulation activate this pathway, evidenced by increase in Nrf2 nuclear translocation, upregulation of HO-1 expression, reduction in oxidative markers, and improvement in cognitive performance. These changes correlate with attenuated neuroinflammation and enhanced antioxidant capacity.^[90]^ Additional research demonstrates that acupuncture at GV23 and GV16 upregulates the Nrf2/HO-1 axis while rebalancing apoptotic factors, collectively preserving neuronal viability in hippocampal regions.^[91]^ The differential effects observed across studies suggest acupuncture’s ability to precisely modulate the Nrf2/ARE/HO-1 pathway according to specific pathological requirements – either suppressing overactivation or enhancing protective responses. This adaptive regulation highlights acupuncture’s potential as a targeted therapy for oxidative stress management in epilepsy.

## 5. Discussion

This study systematically reveals the multi-target regulatory mechanism of acupuncture for epilepsy, providing a new theoretical basis for the modernized interpretation of traditional Chinese medicine therapy. Through integrated analysis, we found that acupuncture can synergistically regulate key signaling pathways such as PI3K/Akt, TLR4, NF-κB, mTOR, MAPK, BDNF/TrkB, and Nrf2/ARE/HO-1, and that a complex network of interactions is formed among these pathways, which together constitute the molecular basis of the antiepileptic therapy of acupuncture. Especially noteworthy is that the present study revealed a specific regulation law of “acupoints-signaling pathways-effects,” which is manifested as follows:

1. Acupuncture treatment of epilepsy is accomplished through the modulation of different signaling pathways, and a single acupuncture point can modulate multiple signaling pathways. For example, acupuncture at the ST36 point in rats can regulate PI3K/Akt, MAPK, BDNF/TrkB, or Nrf2/ARE/HO-1 signaling pathways to exert antiepileptic effects. Acupuncture at the GV20 point in rats can regulate PI3K/Akt, NF-κB, mTOR, BDNF/TrkB, or Nrf2/ARE/HO-1 signaling pathways to exert antiepileptic effects. Acupuncture at the LI11 point in rats can regulate PI3K/Akt, NF-κB, mTOR, BDNF/TrkB, or Nrf2/ARE/HO-1 signaling pathways to exert antiepileptic effects.
2. Acupuncture for epilepsy can regulate the same signaling pathway through different acupuncture points. For example, in the process of controlling the BDNF/TrkB pathway, acupuncture at various points can treat epilepsy through multiple interventional pathways: acupuncture at the ST36 and LI11 points in rats can reduce neuronal apoptosis and restore the plasticity of the hippocampal neuronal synaptic structure to alleviate the cognitive impairment associated with epilepsy; and acupuncture at the HT7 points in rats can focus on decreasing the secretion of pro-inflammatory cytokines to alleviate seizures through anti-inflammatory effects. In addition, acupuncture at different acupoints can also treat epilepsy through the same mechanism of action. Notably, 3 different acupoint pairings – GV20-GV14, LI4-LR3, and ST36-LI11-converge on PI3K/Akt pathway activation despite their anatomical differences. This shared mechanism mediates their antiapoptotic effects in hippocampal neurons, accounting for their observed neuroprotective and antiepileptic properties.
3. Acupuncture with multiple acupuncture points may produce more significant antiepileptic therapeutic effects through the synergistic regulation of signaling pathways. For example, in the Nrf2/ARE/HO-1 signaling pathway, the combination of GV20 and ST36 acupoints activated the pathway more effectively than single acupoint stimulation of ST36, which not only significantly inhibited oxidative stress in the hippocampus, but also reduced neuroinflammation and improved cognitive function. Similarly, in the NF-κB signaling pathway, the combination of GV20 and GV24 points showed a more comprehensive neuroprotective effect than the single-point intervention of GV20, exerting an anti-inflammatory effect by downregulating the NF-κB signaling pathway, and at the same time regulating the expression of apoptotic cytokines, which effectively reduced the neuronal apoptosis and improved the cognitive function significantly.

The current findings establish a robust experimental foundation for understanding acupuncture’s antiepileptic mechanisms while simultaneously informing its clinical optimization. However, several critical knowledge gaps remain that warrant systematic investigation to fully elucidate acupuncture’s therapeutic potential. Two priority research directions emerge from our analysis:

First, the pathway-specific modulation patterns require precise characterization, particularly for complex cascades like mTOR and Nrf2/ARE/HO-1 where acupuncture demonstrates bidirectional regulatory capacity.

Second, quantitative mapping is required for the parameter-effect relationships between acupuncture stimulation and pathway activation. Systematic studies should evaluate: how acupoint selection (single vs combination) influences pathway activation thresholds, how needling depth (muscle layer vs connective tissue) affects signal transduction, how stimulation parameters (intensity, frequency, duration) modulate dose-response curves, and how temporal factors (treatment intervals, cumulative sessions) impact pathway sensitization.

Addressing these questions will enable data-driven optimization of acupuncture protocols, bridging traditional knowledge with modern precision medicine paradigms. Such research promises to not only advance our understanding of acupuncture’s systems-level neuromodulation but also facilitate the development of standardized, evidence-based treatment guidelines for epilepsy management. The integration of these mechanistic insights with clinical outcomes will ultimately position acupuncture as a scientifically-grounded complementary therapy in contemporary epilepsy treatment regimens.

## Acknowledgments

Figures 1–3 were created with BioGDP.com. The authors wish to thank Prof Ming Liu of Heilongjiang University of Chinese Medicine for reading and revising the manuscript carefully.

## Author contributions

**Conceptualization:** Xuan Wang, Weiping Cheng, Qi Zhang.

**Data curation:** Peiyao Meng, Yuxi Jiang.

**Formal analysis:** Peiyao Meng, Wentao Yang, Yuxi Jiang.

**Funding acquisition:** Weiping Cheng, Qi Zhang.

**Supervision:** Xuan Wang, Qi Zhang.

**Writing – original draft:** Peiyao Meng.

**Writing – review & editing:** Xuan Wang, Wentao Yang, Weiping Cheng.
